# Supplementary material for: Nanobodies as novel tools to monitor the mitochondrial fission factor Drp1
Source: Life Sci Alliance. 2024 May 30;7(8):e202402608. doi: 10.26508/lsa.202402608 (PMC11140114; doi:10.26508/lsa.202402608)
Supplement: Supplementary file 1 [file LSA-2024-02608_TableS1.docx]

**Supplementary Table 1**:

| Drp1 Nb | **Amino acid sequence** |
| --- | --- |
| **D7** | QVQLVESGGGLVQPGGSLRLSCVASGFDFSTYSMTWHRQVLGKERELVASITPGGSRTNVADSVKGRFTISRDNAKNMLYLQMDRLKPEDTGMYYCAQGGYYAEPDYWGKGTRVTVSS |
| **D49** | EVQLVESGGGWVQPGGSLRLSCSASGFTFRDYVMTWVRQTPGRGLEWVSAIDGSGSKINYLDSVKGRFTISRDNTQAMLYLQMNSLRPDDTAVYYCARKGRGGLGEGTYWGKGTQVTVSS |
| **D63** | QVQLVESGGDLVQPGGSLKLSCAVSGVSDSLSSFYLMAWHRQAPGKQREMIADIVSGKATYADSVKGRFTISRDNAENTAYLQMNSLKPEDTAVYYCNVLIQNRSTFQSYWGQGTQVTVSS |
| **D187** | HVQLVESGGGLVQAGESLKLSCVASGIPFSSRAMGWYRQTPGKDRELVARISVRGVTFYADSVTGRFAISRDNDRSTLYLQMNSLKSEDTAIYFCAAGDNTQTVLTRPAHWGQGTQVTVSS |
| **D193** | EVQLVDSGGGLVQSGGSLTLACAASVSIDEFPAMTWYRQATGKQRELVGVITKGGATKLADSAKGRFTISRDNAKNMVYLQMTSLKPDDTAVYYCSVPNKVMGWGPDDWWGQGSQVTVSS |
| **D219** | QVQLVESGGGMVQSGGSLSLSCAASGFAFSLYEMSWIRQAQGRGPEWVASINPGSSRTYYTDSVKGRFTISRDNDKNILYLKMNSLKPEDTALYYCARGRTLGIGISSDKGSQVTVSS |
| **D246** | EVQLVESGGGLAQPGGSLRLSCAASGPTDNIYTMGWYRQPPGQSRQFVASVVWSTGVKAYAKFVGGRFRITKDSAKRTVDLQMDSLQPEDTAIYFCNLNDRVTPMSERDYWGQGTPVTVSS |
| **D258** | EVQLQESGGESVQPGGSLRLSCVVSGTTFSTVAMGWYREVPGKKRELVARISSRGVTFYSDSVEGRFTISKDNDKSTLYLQMNSLETEDTAMYFCAAAGNSEVVLFRPKYWGQGTPVTVSS |
