# Supplementary material for: Nanobodies as novel tools to monitor the mitochondrial fission factor Drp1
Source: Life Sci Alliance. 2024 May 30;7(8):e202402608. doi: 10.26508/lsa.202402608 (PMC11140114; doi:10.26508/lsa.202402608)
Supplement: Supplementary file 2 [file LSA-2024-02608_TableS2.docx]

**Supplementary Table 2:**

| **Protein** | **Gene** | **Class A** | **Class B** | **-Log (p-value)** | **Difference** |
| --- | --- | --- | --- | --- | --- |
| Dynamin-1-like protein | DNM1L | + |  | 5.67 | 2.52 |
| Polymerase delta-interacting protein 2 | POLDIP2 |  | + | 3.95 | 1.36 |
| Kinesin-like protein KIF15 | KIF15 |  | + | 5.21 | 1.35 |
| Cation-dependent mannose-6-phosphate receptor | M6PR |  | + | 3.75 | 1.17 |
| Transmembrane emp24 domain-containing protein 1 | TMED1 |  | + | 1.31 | 0.95 |
| 60S ribosomal protein L30 | RPL30 |  | + | 2.38 | 0.93 |
| Vesicle-associated membrane protein 3 | VAMP3 |  | + | 1.29 | 0.86 |
| Neuroplastin | NPTN |  | + | 4.45 | 0.85 |
| Caspase-14 | CASP14 |  | + | 2.66 | 0.84 |
| Protein CutA | CUTA |  | + | 1.18 | 0.80 |
| LanC-like protein 1 | LANCL1 |  | + | 3.35 | 0.80 |
| LysM and putative peptidoglycan-binding domain-containing protein 2 | LYSMD2 |  | + | 1.46 | 0.76 |
| CDGSH iron-sulfur domain-containing protein 1 | CISD1 |  | + | 3.67 | 0.73 |
| Guanine nucleotide-binding protein G(I)/G(S)/G(T) subunit beta-1 | GNB1 |  | + | 2.81 | 0.72 |
| Gamma-glutamylcyclotransferase | GGCT |  | + | 1.15 | 0.72 |
| Microtubule-associated protein 4 | MAP4 |  | + | 1.67 | 0.70 |
| DDB1- and CUL4-associated factor 12 | DCAF12 |  | + | 1.93 | 0.70 |
| Dynein light chain roadblock-type 1;Dynein light chain roadblock-type 2 | DYNLRB1;DYNLRB2 |  | + | 2.17 | 0.69 |
| Hemoglobin subunit beta;Hemoglobin subunit delta | HBB;HBD |  | + | 3.85 | 0.67 |
| Protein canopy homolog 2 | CNPY2 |  | + | 1.27 | 0.66 |
| Mitochondrial import receptor subunit TOM20 homolog | TOMM20 |  | + | 2.23 | 0.66 |
| Histone deacetylase 6 | HDAC6 |  | + | 2.22 | 0.65 |
| Protein bicaudal D homolog 2 | BICD2 |  | + | 4.28 | 0.64 |
| Sodium-coupled neutral amino acid transporter 2 | SLC38A2 |  | + | 1.35 | 0.63 |
| Cytochrome c oxidase subunit 5A | COX5A |  | + | 1.17 | 0.63 |
| Protein YIPF4 | YIPF4 |  | + | 2.60 | 0.62 |
| Lysosome membrane protein 2 | SCARB2 |  | + | 1.82 | 0.62 |
| Cytochrome b5 type B | CYB5B |  | + | 1.26 | 0.62 |
| Synaptogyrin-1 | SYNGR1 |  | + | 1.41 | 0.61 |
| DNA damage-binding protein 1 | DDB1 |  | + | 3.26 | 0.61 |
| Transmembrane protein 181 | TMEM181 |  | + | 1.97 | 0.59 |
| MICOS complex subunit MIC19 | CHCHD3 |  | + | 2.75 | 0.59 |
| DDRGK domain-containing protein 1 | DDRGK1 |  | + | 1.70 | 0.55 |
| Manganese-transporting ATPase 13A1 | ATP13A1 |  | + | 2.55 | 0.54 |
| Coxsackievirus and adenovirus receptor | CXADR |  | + | 1.80 | 0.53 |
| ATP synthase subunit d, mitochondrial | ATP5H |  | + | 1.97 | 0.51 |
