# Supplementary material for: Nanobodies as novel tools to monitor the mitochondrial fission factor Drp1
Source: Life Sci Alliance. 2024 May 30;7(8):e202402608. doi: 10.26508/lsa.202402608 (PMC11140114; doi:10.26508/lsa.202402608)
Supplement: Supplementary file 3 [file LSA-2024-02608_TableS3.docx]

**Supplementary Table 3:**

| **Protein** | **Gene** | **Class A** | **Class B** | **-Log(P-value)** | **Difference** |
| --- | --- | --- | --- | --- | --- |
| Dynamin-1-like protein | DNM1L | + |  | 5.61 | 2.55 |
| Peroxisomal multifunctional enzyme type 2 | HSD17B4 | + |  | 3.85 | 1.95 |
| GDP-L-fucose synthase | TSTA3 | + |  | 5.71 | 1.30 |
| Hydroxysteroid dehydrogenase-like protein 2 | HSDL2 | + |  | 3.11 | 1.29 |
| Protein CutA | CUTA | + |  | 3.69 | 1.22 |
| Transmembrane emp24 domain-containing protein 1 | TMED1 | + |  | 3.39 | 1.20 |
| Mitochondrial import inner membrane translocase subunit Tim10 B | TIMM10B | + |  | 4.50 | 1.17 |
| Bifunctional lysine-specific demethylase and histidyl-hydroxylase NO66 | NO66 |  | + | 2.95 | 1.05 |
| Cystatin-A | CSTA |  | + | 1.31 | 0.96 |
| Tight junction protein ZO-1 | TJP1 |  | + | 3.21 | 0.92 |
| Importin-11 | IPO11 |  | + | 1.68 | 0.92 |
| Exportin-7 | XPO7 |  | + | 2.87 | 0.92 |
| Vesicle-associated membrane protein 3 | VAMP3 |  | + | 1.34 | 0.87 |
| Caspase-14 | CASP14 |  | + | 1.09 | 0.86 |
| 60S ribosomal protein L30 | RPL30 |  | + | 2.24 | 0.85 |
| E3 ubiquitin-protein ligase TRIM9 | TRIM9 |  | + | 2.56 | 0.78 |
| Mitochondrial import receptor subunit TOM20 homolog | TOMM20 |  | + | 2.53 | 0.77 |
| ATP synthase subunit beta | ATP5B |  | + | 1.39 | 0.76 |
| Apoptosis-inducing factor 1 | AIFM1 |  | + | 3.21 | 0.73 |
| CDGSH iron-sulfur domain-containing protein 1 | CISD1 |  | + | 3.82 | 0.71 |
| Protein-L-isoaspartate O-methyltransferase domain-containing protein 2 | PCMTD2 |  | + | 1.88 | 0.70 |
| Serine/threonine-protein kinase 3 | STK3 |  | + | 2.83 | 0.69 |
| STE20/SPS1-related proline-alanine-rich protein kinase | STK39 |  | + | 5.23 | 0.69 |
| LIM domain and actin-binding protein 1 | LIMA1 |  | + | 1.52 | 0.68 |
| Adenylate kinase isoenzyme 6 | TAF9;AK6 |  | + | 3.90 | 0.67 |
| MICOS complex subunit MIC19 | CHCHD3 |  | + | 2.91 | 0.63 |
| Transcription elongation factor B polypeptide 2 | TCEB2 |  | + | 2.20 | 0.62 |
| Histone deacetylase 6 | HDAC6 |  | + | 2.07 | 0.58 |
| Transcription elongation factor B polypeptide 1 | TCEB1 |  | + | 2.39 | 0.57 |
| Protein-L-isoaspartate(D-aspartate) O-methyltransferase | PCMT1 |  | + | 3.82 | 0.57 |
| Ubiquitin carboxyl-terminal hydrolase isozyme L5 | UCHL5 |  | + | 3.43 | 0.56 |
| Protein-L-isoaspartate O-methyltransferase domain-containing protein 1 | PCMTD1 |  | + | 3.03 | 0.55 |
