# Supplementary material for: Nanobodies as novel tools to monitor the mitochondrial fission factor Drp1
Source: Life Sci Alliance. 2024 May 30;7(8):e202402608. doi: 10.26508/lsa.202402608 (PMC11140114; doi:10.26508/lsa.202402608)
Supplement: Supplementary file 4 [file LSA-2024-02608_TableS4.docx]

**Supplementary Table 4:**

| **Protein** | **Gene** | **Class A** | **Class B** | **-Log(P-value)** | **Difference** |
| --- | --- | --- | --- | --- | --- |
| **D7** | | | | | |
| Dynamin-1-like protein | DNM1L | + |  | 4,40 | 3,31 |
| Dynamin-1-like protein | DNM1L | + |  | 4,55 | 2,34 |
| Polymerase delta-interacting protein 2 | POLDIP2 |  | + | 4,52 | 1.17 |
| Kinesin-like protein KIF15 | KIF15 |  | + | 4,99 | 1,86 |
| **D63** | | | | | |
| Dynamin-1-like protein | DNM1L | + |  | 4,28 | 3,09 |
| Dynamin-1-like protein | DNM1L |  | + | 2,98 | 0,92 |
| Dynamin-1-like protein | DNM1L |  | + | 3,55 | 1,95 |
| Kelch-like protein 13;Kelch-like protein 9 | KLHL13;KLHL9 |  | + | 3.35 | 1.09 |
| GDP-L-fucose synthase | TSTA3 |  | + | 4,53 | 1,11 |
| Protein-L-isoaspartate O-methyltransferase domain-containing protein 1 | PCMTD1 |  | + | 4.34 | 1.24 |
| Mitochondrial import inner membrane translocase subunit Tim10 B | TIMM10B |  | + | 2,84 | 1,03 |
