# Supplementary material for: Nanobodies as novel tools to monitor the mitochondrial fission factor Drp1
Source: Life Sci Alliance. 2024 May 30;7(8):e202402608. doi: 10.26508/lsa.202402608 (PMC11140114; doi:10.26508/lsa.202402608)
Supplement: Supplementary file 5 [file LSA-2024-02608_TableS5.docx]

**Supplementary Table 5**

| **Protein** | **Gene** | **Class A** | **Class B** | **-Log(P-value)** | **Difference** |
| --- | --- | --- | --- | --- | --- |
| **D7** | | | | | |
| Dynamin-1-like protein | DNM1L |  | + | 3.69 | 3.02 |
| Dynamin-1-like protein | DNM1L |  | + | 3.33 | 1.37 |
| Polymerase delta-interacting protein 2 | POLDIP2 |  | + | 3,642 | 1,31 |
| Kinesin-like protein KIF15 | KIF15 |  | + | 3,35 | 1,27 |
| **D63** | | | | | |
| Dynamin-1-like protein | DNM1L |  | + | 3,63 | 2,62 |
| GDP-L-fucose synthase | TSTA3 |  | + | 5,00 | 0,90 |
| Enhancer of mRNA-decapping protein 3 | EDC3 |  | + | 4.50 | 0.82 |
| Kelch-like protein 13;Kelch-like protein 9 | KLHL13;KLHL9 |  | + | 3.35 | 1.09 |
| Lymphokine-activated killer T-cell-originated protein kinase | PBK |  | + | 4.50 | 0.78 |
