# Supplementary material for: Nanobodies as novel tools to monitor the mitochondrial fission factor Drp1
Source: Life Sci Alliance. 2024 May 30;7(8):e202402608. doi: 10.26508/lsa.202402608 (PMC11140114; doi:10.26508/lsa.202402608)
Supplement: Supplementary file 6 [file LSA-2024-02608_TableS6.docx]

**Supplementary Table 6:**

| **Name** | **Sequence 5’- 3’** | **purpose** |
| --- | --- | --- |
| CALL001 | GTCCTGGCTGCTCTTCTACAAGG | Nb library generation |
| CALL002 | GGTACGTGCTGTTGAACTGTTCC |  |
| FR1-1 | CATGGCNSANGTGCAGCTGGTGGANTCNGGNGG |  |
| FR1-2 | CATGGCNSANGTGCAGCTGCAGGANTCNGGNGG |  |
| FR1-3 | CATGGCNSANGTGCAGCTGGTGGANAGYGGNGG |  |
| FR1-4 | CATGGCNSANGTGCAGCTGCAGGANAGYGGNGG |  |
| FR1-ext1 | GTAGGCCCAGCCGGCCATGGCNSANGTGCAGCTGGTGG |  |
| FR1-ext2 | GTAGGCCCAGCCGGCCATGGCNSANGTGCAGCTGCAGGA |  |
| FR4-1 | GATGCGGCCGCNGANGANACGGTGACCNGNRYNCC |  |
| FR4-2 | GATGCGGCCGCNGANGANACGGTGACCNGNGANCC |  |
| FR4-3 | GATGCGGCCGCNGANGANACGGTGACCNGRCTNCC |  |
| FR4-4 | GATGCGGCCGCRCTNGANACGGTGACCNGNRYNCC |  |
| FR4-5 | GATGCGGCCGCRCTNGANACGGTGACCNGNGANCC |  |
| FR4-6 | GATGCGGCCGCRCTNGANACGGTGACCNGRCTNCC |  |
|  |  |  |
| bivD7-1 | GAAACGTCTCAACTCTCAGGTGCAGCTGGTGGAGAG | Cloning of bivNbs |
| bivD7-2 | CACCACCGCCAGATCCACCGCCACCTGATCCTCCGCCTCCTGAGGAGACGGTGACCCG |  |
| bivD7-3 | GGTGGATCTGGCGGTGGTGGAAGTGGTGGCGGAGGTAGTCAGGTGCAGCTGGTGGAGAG |  |
| downEcoRI-rev | GTTGTAAAACGACGGCCAGTG |  |
| bivNshort_for | GAAACGTCTCAACTCTCAGGTGC |  |
| bivD63forN | GAAACGTCTCAACTCTCAAGTGCAGCTGGTGGAGTC |  |
| bivNtermGS-rev | CACCACCGCCAGATCCACCGCCACCTGATCCTCCGCCTCCGCTGCTAACGGTAACCTG |  |
| bivD63forC | GGTGGATCTGGCGGTGGTGGAAGTGGTGGCGGAGGTAGTCAAGTGCAGCTGGTGGAGTC |  |
| bivD63_fusion | GAAACGTCTCAACTCTCAAGTGC |  |
|  |  |  |
| Drp1_GTPaseFor | GTGGTGGCGGAGGTAGCATGGAGGCGCTAATTCCTGTC | GFP-Drp1 generation |
| Drp1_C_term_rev | CTAGATCCGGTGGATCCTCACCAAAGATGAGTCTCCCG |  |
|  |  |  |
| Drp1_VD_rev | CTAGATCCGGTGGATCCTCATTTTCGTGCAACAGGAAC | generation of domain deletion constructs |
| Drp1_MD_rev | CTAGATCCGGTGGATCCTCAAAAGTCTGGATGTTTTGTG |  |
| Drp1_GTPaseRev | CTAGATCCGGTGGATCCTCATTGGAGTAAAGTAGCACTTTTATC |  |
