# Supplementary material for: Nanobodies as novel tools to monitor the mitochondrial fission factor Drp1
Source: Life Sci Alliance. 2024 May 30;7(8):e202402608. doi: 10.26508/lsa.202402608 (PMC11140114; doi:10.26508/lsa.202402608)
Supplement: Supplementary file 7 [file LSA-2024-02608_TableS7.docx]

**Supplementary Table 7:**

| **construct** | **origin** |
| --- | --- |
| pHEN4 | (Arbabi Ghahroudi M et al, 1997) |
| pHEN6C | (Rothbauer U et al, 2008) |
| D7 in pHEN6C | This study |
| D63 in pHEN6C | This study |
| pCDNA3.4 | Addgene |
| bivD7 in pCDNA3.4 | This study |
| bivD63 in pCDNA3.4 | This study |
| pTagRFP | (Panza P et al, 2015) |
| D7 in pTagRFP | This study |
| D63 in pTagRFP | This study |
| pEGFP-Ubi-R-3xGS-tagRFP | (Keller BM et al, 2018) |
| D7 in pEGFP-Ubi-R-3xGS-tagRFP | This study |
| D63 in pEGFP-Ubi-R-3xGS-tagRFP | This study |
| AcGFP-Drp1 | (Jenner A et al, 2022) |
| pEGFP-N1 | Clontech |
| Drp1 in pEGFP-N1 | This study |
| GTPase-MD-VD in pEGFP-N1 | This study |
| GTPase-MD in pEGFP-N1 | This study |
| GTPase in pEGFP-N1 | This study |
| pEGFP-C1 | Clontech |
| Pep-Cb in pTagRFP | (Traenkle B et al, 2020) |
| BC2T-GFP-NLS in pEGFPN1 | (Virant D et al, 2018) |
| pTYB2 | (Jenner A et al, 2022) |
