# Supplementary material for: Nanobodies as novel tools to monitor the mitochondrial fission factor Drp1
Source: Life Sci Alliance. 2024 May 30;7(8):e202402608. doi: 10.26508/lsa.202402608 (PMC11140114; doi:10.26508/lsa.202402608)
Supplement: Supplementary file 8 [file LSA-2024-02608_TableS8.docx]

**Supplementary Table 8:**

| **target** | **animal** | **clone** | **conjugation** | **manufacturer** | **catalog number** |
| --- | --- | --- | --- | --- | --- |
| Drp1 | rabbit | D6C7 | - | Cell Signaling Technology | #8570 |
| GAPDH | rabbit | polyclonal | - | Invitrogen | PA1-987 |
| GFP | rat | 3H9 | - | ChromoTek | - |
| TagRFP | rabbit | polyclonal | - | Evrogen | AB234 |
| M13 | mouse | monoclonal | HRP | GE Healthcare | 27-9421-01 |
| rabbit | donkey | polyclonal | AF647 | Invitrogen | #A-31573 |
| rabbit | goat | polyclonal | AF488 | Invitrogen | #A-11008 |
| rat | donkey | polyclonal | AF647 | Invitrogen | #A-21247 |
| alpaca | goat | polyclonal | Cy5 | Jackson Immunoresearch | 128-175-232 |
